# Running throughout Middle-Age Keeps Old Adult-Born Neurons Wired

**DOI:** 10.1523/ENEURO.0084-23.2023

**Published:** 2023-05-17

**Authors:** Carmen Vivar, Ben Peterson, Alejandro Pinto, Emma Janke, Henriette van Praag

**Affiliations:** 1 Department of Physiology, Biophysics and Neuroscience, Centro de Investigación y de Estudios Avanzados del Instituto Politécnico Nacional, Mexico City 07360, Mexico; 2 National Institute on Aging, Baltimore, MD 21224; 3 Department of Biomedical Sciences, Charles E. Schmidt College of Medicine, and Stiles-Nicholson Brain Institute, Florida Atlantic University, Jupiter, FL 33458

**Keywords:** aging, dentate gyrus, interneurons, adult neurogenesis, perirhinal cortex, running

## Abstract

Exercise may prevent or delay aging-related memory loss and neurodegeneration. In rodents, running increases the number of adult-born neurons in the dentate gyrus (DG) of the hippocampus, in association with improved synaptic plasticity and memory function. However, it is unclear whether adult-born neurons remain fully integrated into the hippocampal network during aging and whether long-term running affects their connectivity. To address this issue, we labeled proliferating DG neural progenitor cells with retrovirus expressing the avian TVA receptor in two-month-old sedentary and running male C57Bl/6 mice. More than six months later, we injected EnvA-pseudotyped rabies virus into the DG as a monosynaptic retrograde tracer, to selectively infect TVA expressing “old” new neurons. We identified and quantified the direct afferent inputs to these adult-born neurons within the hippocampus and (sub)cortical areas. Here, we show that long-term running substantially modifies the network of the neurons generated in young adult mice, upon middle-age. Exercise increases input from hippocampal interneurons onto “old” adult-born neurons, which may play a role in reducing aging-related hippocampal hyperexcitability. In addition, running prevents the loss of adult-born neuron innervation from perirhinal cortex, and increases input from subiculum and entorhinal cortex, brain areas that are essential for contextual and spatial memory. Thus, long-term running maintains the wiring of “old” new neurons, born during early adulthood, within a network that is important for memory function during aging.

## Significance Statement

Exercise benefits brain function; however, the underlying mechanisms remain unclear. We show that long-term running increases hippocampal neurogenesis and modifies the network of new neurons that were born in young adult mice, in a manner that optimally supports memory function at middle age.

## Introduction

Worldwide, the proportion of older adults is expanding more than any other age group (Humphreys, 2012). Aging is often accompanied by cognitive decline, and among the first affected structures are the hippocampus and adjacent cortices (Scoville and Milner, 2000; Lester et al., 2017), brain areas essential for learning and memory. An initial symptom is an impaired ability to distinguish between highly similar stimuli and events, called pattern separation (Marr, 1971). Deficits therein are associated with reduced hippocampal volume and degradation of synaptic connectivity between (peri)entorhinal cortex and the hippocampus (Yassa et al., 2010, 2011; Burke et al., 2011). Increasing evidence indicates that physical activity can delay or prevent such structural and functional reductions in older adults (Erickson et al., 2011; Voss et al., 2013, 2019; Duzel et al., 2016; Gaitán et al., 2021; Barnes et al., 2022). The underlying mechanisms are considered to involve central and systemic changes that result in increased levels of hippocampal neurotrophins, neurotransmitters, vascularization, and attenuated inflammation (Huuha et al., 2022; Gao et al., 2023). Moreover, in rodents, hippocampal neurogenesis, a process proposed to make unique contributions to spatial and contextual learning (Kent et al., 2016; Stevenson et al., 2020), is substantially increased by voluntary wheel running (van Praag et al., 1999; Vivar et al., 2013; Voss et al., 2019). The adult-born neurons in the dentate gyrus (DG) are part of an extensive (sub)cortical network (Vivar et al., 2012, 2016), and their increment is positively associated with improved pattern separation ability (Creer et al., 2010; Sahay et al., 2011) and spatial memory function (van Praag et al., 1999; Voss et al., 2013). However, it remains unclear, whether and how exercise affects the connectivity of new neurons during aging.

Here, we focused on the effects of long-term running on the network of new neurons that were generated in young adult mice, at middle-age. Considering that adult neurogenesis is strongly diminished with aging (Seki and Arai, 1995), the neurons born in young adults may have more functional relevance than those generated in middle-age or old-age. Conversely, research suggests that adult-born neurons are only temporarily important, during the so-called “critical period” at approximately three to six weeks of cell age, when they can transiently display increased synaptic plasticity (Schmidt-Hieber et al., 2004; Kee et al., 2007; Danielson et al., 2016) and modifications in neural circuitry (Bergami et al., 2015). Even so, new neurons remain present for many months (Dayer, 2003; Marlatt et al., 2012), and it is unclear whether those born in early adulthood remain integrated into neural networks, and whether their circuitry is modifiable by physical activity in middle age. To begin to address this question we used a unique rabies virus-based circuit tracing approach (Vivar et al., 2012, 2016) with a long time interval between the initial labeling of new neurons and subsequent input identification. Specifically, dividing hippocampal neural progenitor cells were labeled with retrovirus (expressing a fluorescent reporter, avian TVA receptor and rabies glycoprotein) in young adult mice. Adult-born neuron network evaluation took place months later, when mice were middle-aged, by using avian EnvA pseudotyped rabies virus as a monosynaptic retrograde tracer.

Our research shows that the afferent network of adult-born neurons is drastically changed by long-term running. Intrahippocampally, input from dorsal area CA3 pyramidal cells (PYRs), long-range interneurons (INTs; areas CA3 and CA1), and the subiculum are increased. In addition, running upregulates the connectivity from the caudomedial entorhinal cortex (CEnt), which conveys spatial information (Marks et al., 2021), and prevents the loss of input from perirhinal cortex (PRH), a brain area essential for spatial discrimination (Bussey et al., 2005; Suzuki and Naya, 2014). Altogether, our findings show that long-term running wires “old” new neurons, born during early adulthood, into a network that is relevant to the maintenance of episodic memory encoding during aging.

## Materials and Methods

### Animals

Male C57Bl6 mice (The Jackson Laboratory) five to six weeks old (*n *=* *18) were individually housed and randomly assigned to control standard (CON) or voluntary wheel running (RUN) conditions, in 12/12 h light/dark cycle with food and water *ad libitum*. Voluntary exercise was performed in a silent spinner running wheel (11.5 cm in diameter). Running distance was monitored as described previously (Creer et al., 2010). Mice were maintained according to the National Institutes of Health guidelines, and protocols for procedures were approved by the National Institute on Aging Institutional Animal Care and Use Committee.

### Viral vector production

Retroviral vector expressing nuclear green fluorescent protein (GFP), avian TVA receptor and rabies virus glycoprotein (Rgp) driven by the neuron-specific synapsin (SYN) promoter (RV-SYN-GTRgp), and EnvA-pseudotyped Δgp-mCherry rabies virus (EnvA-ΔG-MCh), were produced as previously described (Wickersham et al., 2007; Vivar et al., 2012). Specifically, retrovirus RV-SYN-GTRgp was produced by transient transfection (Lipofectamine 2000, Invitrogen) of the vector (7.5 μg), CMVGagPol (5 μg), and CMV-VSVG (2.5 μg) in 90% confluent 293T cells. Virus-containing supernatant was harvested 36 h later filtered and concentrated by ultracentrifugation. Virus titers were estimated to be ∼10 × E8 i.u. ml^−1^ by serial dilution into 293T cells. To produce EnvA-ΔG-MCh, a glycoprotein-gene-deleted rabies virus vector (Δgp-mCherry) was generated in which a mCherry (MCh) reporter gene was inserted into the locus encoding the rabies virus glycoprotein (kindly provided by E. Callaway, Salk Institute). The helper cell line, BHK-EnvARGCD, was infected with Δgp-mCherry, to produce EnvA pseudotyped rabies virus. Supernatants containing Δgp-mCherry rabies virus pseudotyped with EnvA were harvested 5 d later, filtered and concentrated by ultracentrifugation. Rabies virus titer was estimated to be ∼1.2 × 10 E7 i.u. ml^−1^ and diluted for use to ∼4 × 10E6 i.u. ml^−1^.

### Stereotaxic surgery

After 3 d of housing in their respective conditions, mice were anesthetized (Avertin 0.4 mg g^−1^, i.p.) and stereotaxic surgery was performed to deliver 1 μl of RV-SYN-GTRgp retrovirus into the right dorsal and ventral dentate gyrus (DG) using spatial coordinates relative to bregma as follows: dorsal DG, anterior-posterior (AP) = −2.10 mm; medial-lateral (ML) = 1.9 mm; dorso-ventral (DV) = −2.20 mm, and ventral DG, AP = −3.10 mm; ML = 2.8 mm; DV = −3.20 mm. These coordinates were modified from the mouse brain atlas (Paxinos and Franklin, 2007) and adjusted for mice aged five to six weeks old at the time of injection. After six to nine months, the retrovirus-injected mice were anesthetized (Avertin 0.4 mg g^−1^, i.p.) and injected with 1 μl of rabies virus EnvA-ΔG-MCh into the same dorsal and ventral DG locations. Seven days thereafter, animals were given an overdose of isofluorane anesthetic (Abbott) and perfused transcardially with 0.9% saline at room temperature (RT) followed by cold 4% paraformaldehyde in 0.1 m PBS. Brains were removed and postfixated for 24 h. Brain tissue was equilibrated in 30% sucrose and sequential horizontal sections (40 μm) were taken using a freezing microtome (HM450, ThermoFisher) throughout the dorso-ventral extent of the brain. Brain sections were stored in 96-well plates containing phosphate-buffered glycerol at −20°C until further analysis.

### Immunohistochemistry

To identify cell types innervating the mature adult-born granule cells, labeling was conducted in a 1:6 series (240 μm apart) of horizontal sections (40 μm) through the dorso-ventral extent of the brain. Sections were stained for GFP (chicken polyclonal, 1:1000, catalog #GFP-1010, Aves Labs; RRID: AB_2307313) and RFP (rabbit polyclonal, 1:1000, Rockland Labs, catalog #600-401-379; RRID: AB_2209751) and corresponding fluorescent secondary antibody (donkey anti-chicken Alexa Fluor 488, 1:500, catalog #703-545-155, Jackson ImmunoResearch; RRID: AB_2340375; donkey anti-rabbit CY3 1:250, Jackson ImmunoResearch, catalog #705-165-147; RRID: AB_2307351) as described (Vivar and van Praag, 2022). Nuclei were visualized with DAPI (1:20,000, catalog #D21490, Invitrogen).

### Imaging and cell counts

Old adult-born granule cells in the dentate gyrus were identified by nuclear expression of GFP. Only old adult-born granule cells with dual-infection (retrovirus and rabies virus) expressing nuclear GFP and cytoplasmic MCh are considered to be the origin of the trans-synaptic tracing, which were called “starter cells” (SC). Presynaptic traced cells (TC) located throughout the brain were identified by the expression of MCh only.

To quantify the number of starter cells (GFP^+^-MCh^+^) and traced cells (MCh^+^ only) in the dentate gyrus, confocal images of the dentate gyrus (FV 1000MPE, Olympus), fifteen to eighteen z-planes at 1-μm intervals, were taken at 20× (∼20–24 brain slices per animal, only 10–13 slices contain dentate gyrus area). To evaluate the traced cells in the other brain areas, all the sections (∼20–24 per animal) were imaged at 4× using a fluorescent microscope (BX51, Olympus). Sections were reconstructed by stitching the images using CorelDraw. After reconstruction, sections were matched to the mouse brain atlas (Paxinos and Franklin, 2007) to determine the dorso-ventral distance from bregma and the brain area where the traced cells were found. Next, higher magnification images using a 10× objective (BX51, Olympus) were taken to allow for a detailed quantification of the traced cells. Starter and traced cells were classified and counted by an experimenter who was blinded to the group identity of the samples. Traced cells were counted ipsilateral and contralateral to the side of the viral injections.

The total starter or traced cell numbers were obtained by multiplying by six. Only mouse brains with >40% of starter cells (GFP^+^-MCh^+^) from the total number of old adult-born granule cells (GFP^+^ only) were taken for tracing analysis (four of nine mice of each group). The total number of old adult-born granule cells was calculated by the sum of single-labeled (GFP^+^ only) and double-labeled cells (GFP^+^-MCh^+^). For the analysis of the dorso-ventral distribution of the old adult-born granule cells expressing GFP and the starter cells in the dentate gyrus, the brain sections were matched to the mouse brain atlas, and were divided into dorsal, intermediate, and ventral regions according to the following coordinates (mm from bregma): dorsal: −1.24 to −2.16; intermediate: −2.36 to −2.96; ventral; −3.16 to −4.12. The total number of GFP^+^ and starter cells was calculated as the sum of cells by region (dorsal, intermediate, and ventral). The number of cells from the dorsal, intermediate and ventral regions was compared between control and running groups.

#### Intrahippocampal cells

In the dentate gyrus, the traced cells expressing MCh only were identified as mature granule cells (mGCs), mossy cells (MCs), interneurons, or astrocytes (ASs) based on their location and morphological characteristics. The mature granule cells have an elliptical cell body localized in the granule cell layer (GCL) with a characteristic cone-shaped tree of spiny apical dendrites (Amaral and Lavenex, 2007). Mossy cells were identified by their location in the hilus and their characteristic large spines covering all of its proximal dendrites called thorny excrescences (Amaral and Lavenex, 2007). Dentate gyrus interneurons were identified based on the location of their soma as follows. (1) Interneurons of the molecular layer (ML) if their soma were positioned in the ML. These are typically MOPP cells with a round soma and two primary dendrites emerging from the cell body that give rise to several secondary dendrites fanning out radially into the ML. (2) Interneurons of the granule cell layer if their somata were located in the granule cell layer-hilus border. They are a mix of basket, axo-axonic, HIPP and HICAP cells. Basket cells have pyramidally-shaped soma with prominent apical and basal dendrites emerging from the soma. Axo-axonic cells typically have a dendritic tree with a tuft of several radially running branches. HIPP cells have a large fusiform soma with branches profusely developing into the dendritic tree, and HICAP cells typically have a triangular cell body with primary dendrites from multipolar origins. (3) Hilar interneurons if their somata were located in the hilus. They are a mix of, HICAP, HIPP, and neurogliaform cells (Freund and Buzsáki, 1996; Booker and Vida, 2018).

In the CA1–CA3 areas of the hippocampus, traced interneurons were identified by their morphological characteristics according to the location of their soma and morphology (e.g., axo-axonic, basket cells, bistratified or O-LM cells; Freund and Buzsáki, 1996; Booker and Vida, 2018). CA interneurons are located in the strata oriens, radiatum, lacunosum-moleculare, and lucidum, as well as on the borders of the pyramidal cell layer. Traced pyramidal cells were identified by their pyramidal cell body, their basal and apical dendritic tree that extends into the stratum oriens and hippocampal fissure, respectively (Amaral and Lavenex, 2007).

#### Cortical neurons

Traced entorhinal cortex cells were determined to be in the lateral entorhinal cortex (LEC), perirhinal cortex (PRH), medial entorhinal cortex (MEC), and caudomedial entorhinal cortex (CEnt) using defined parameters (Dolorfo and Amaral, 1998; van Groen, 2001). The remainder of the traced cells were identified by their location in the brain based on the mouse brain atlas (Paxinos and Franklin, 2007).

Photomicrographs for figures were prepared using a parallel series of sections from the same brains used for quantitative analysis.

### Statistical analysis

GraphPad Prism 8 was used for statistical analyses. Comparisons between control and running groups were performed with a Student’s *t* test. Two-way ANOVA with repeated measures (Treatment × Region) followed by *post hoc* Tukey’s multiple comparison test was used for regional distribution analyses. CON versus RUN pairwise comparison for each region was performed when an interaction between factors was identified. Two sample Kolmogorov–Smirnov nonparametric test was used for CON versus RUN comparison when one of the groups presented only ZERO as values. Running distance over time was analyzed with linear regression. A significance level of 0.05 was set for all analyses. All values are shown as mean ± SEM.

## Results

### Running promotes sparse connectivity onto old adult-born neurons

**Figure 1.**
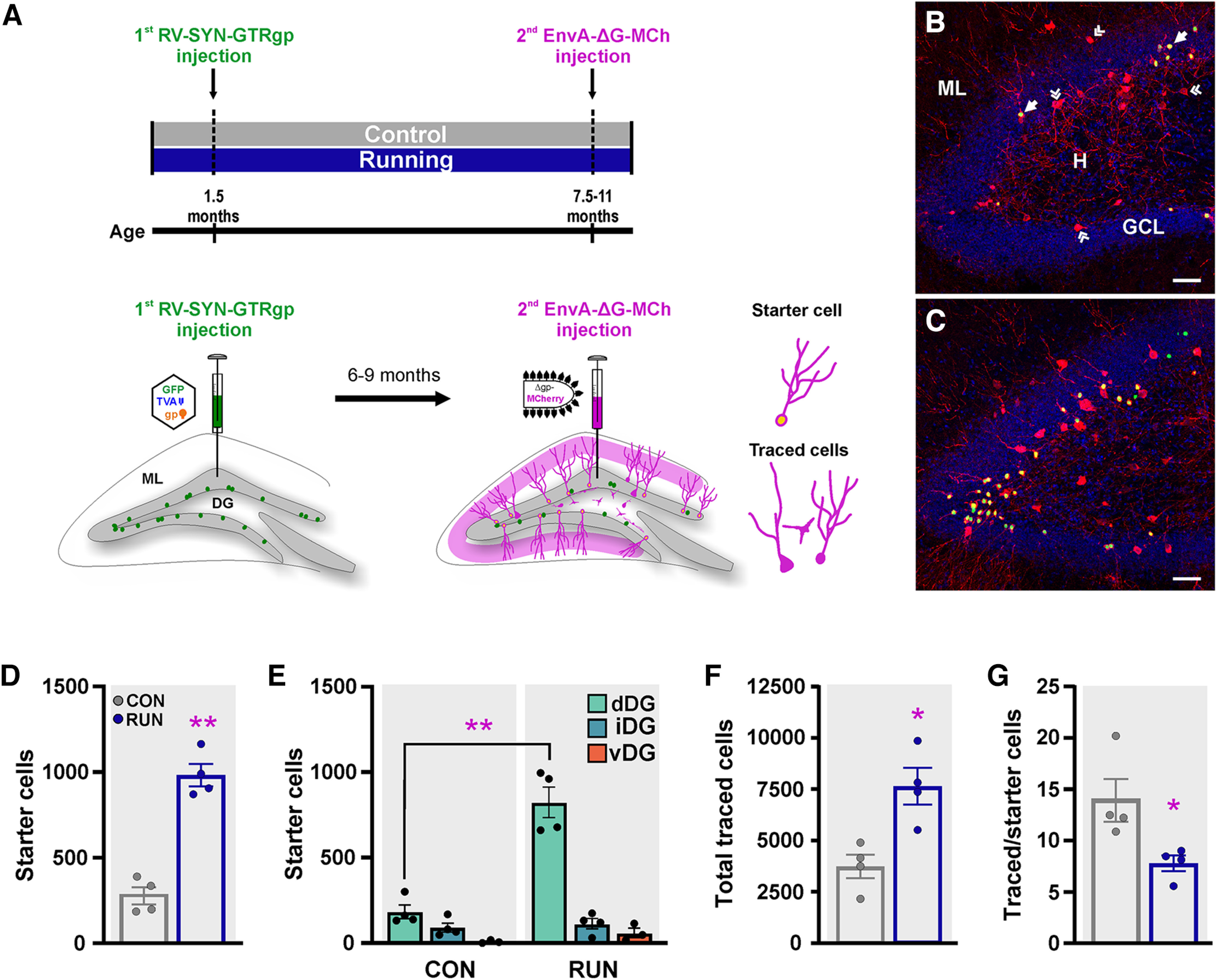
Running increases old adult-born neuron survival and modifies their afferent input in middle-aged mice. ***A***, Timeline of the experiment. Adult young control (CON; *n *=* *4) and runner (RUN; *n *=* *4) mice (1.5 months old) were injected with retrovirus expressing nuclear GFP, avian TVA receptor, and rabies glycoprotein (RV-SYN-GTRgp; first injection) into the dentate gyrus (DG) to label proliferating neural progenitor cells. Mice were housed in either control or voluntary running conditions for six to nine months. Thereafter, mice were injected with EnvA-pseudotyped rabies virus expressing MCh (EnvA-ΔG-MCh, second injection) into the same sites to trace the inputs to the old adult-born neurons. Double infected (GFP^+^ + MCh^+^ = yellow nuclei) cells are adult-born neurons from which the tracing originated, termed “starter cells,” while cells synaptically connected to old adult-born neurons expressing MCh (red) only are termed “traced cells.” ***B***, ***C***, Photomicrographs of the dorsal DG showing starter (⬆) and traced («) cells in hippocampal sections derived from (***B***) CON and (***C***) RUN mice. Nuclei were stained with DAPI (blue). Scale bar: 50 μm. ***D***, Long-term running increases the number of starter cells (*t*_(6)_ = 8.514; *p *=* *0.0001). ***E***, Distribution analysis showed that running increased starter cell number in the dorsal (dDG) but not intermediate (iDG) or ventral (vDG) dentate gyrus (*F*_(2,12)_ = 25.04, *p *<* *0.0001). ***F***, The total traced cell number is increased in RUN as compared with CON mice (*t*_(6)_ = 3.676, *p *=* *0.0104). ***G***, The connectivity index measured as the traced/starter cell ratio is significantly reduced by long-term running (*t*_(6)_ = 2.790, *p *=* *0.0316). Statistical scores are listed in Extended Data Table 1-1. See Extended Data Figure 1-1 for distance run, and GFP^+^ adult-born neuron numbers. Data are means ± SEM *p <* *0.05, **p <* *0.0001. H, hilus; GCL, granule cell layer; ML, molecular layer.

To begin to understand the functional significance of the new granule cells born during early adulthood in the aging brain [“old” new granule cells (OnGC)], we analyzed their neuronal network. Specifically, we used the rabies-virus tracing system to label the first-order presynaptic neurons to OnGC in control and long-term running mice. The monosynaptic TVA-EnvA tracing system has been described in greater detail in previous publications (Wickersham et al., 2007; Vivar et al., 2012; Vivar and van Praag, 2013, 2022) and is described briefly here. First, retrovirus (RV-SYN-GTRgp) expressing nuclear green fluorescent protein (GFP), the TVA receptor, and rabies glycoprotein (Rgp) under control of the neuron-specific synapsin promoter was injected into the dorsal and ventral DG (first injection) of young adult control (*n *=* *9) and runner (*n *=* *9) mice to label proliferating neural progenitor cells that become new neurons over weeks (Fig. 1*A*). Mice were kept in their respective control or running conditions for six to nine months. Mice ran on average a total of 3200 ± 182 km during the entire experiment. There was a significant reduction in distance run over time (Extended Data Fig. 1-1*A*).

10.1523/ENEURO.0084-23.2023.f1-1 Extended Data Figure 1-1 Running increases the survival of old adult-born neurons born during early adulthood. ***A***, Long-term running distance, there is a significant reduction in monthly distance run over time in male C57Bl/6 mice (*n *= 9; *F*_(1,7)_ = 12.86, *p *<* *0.0089). ***B***, ***C***, Photomicrographs of the dorsal dentate gyrus showing GFP^+^ cells from control (CON; ***B***) and long-term running mice (RUN; ***C***). Scale bar: 50 μm. Nuclei were stained with DAPI (blue). ***D***, Long-term running increases the number of total GFP^+^ cells in the dentate gyrus compared to control middle-aged mice (*t*_(6)_ = 5.196, *p *=* *0.002; CON, *n *=* *4; RUN, *n *=* *4). ***E***, Dorsal to ventral distribution analysis shows that running increases the number of GFP^+^ cells in the dorsal (dDG) but not intermediate (iDG) or ventral (vDG) dentate gyrus (*F*_(2,12)_ = 8.64, *p *<* *0.0047). ***F***, The proportion of double-labeled cells (starter cells) with respect to the total number of GFP^+^ cells is similar between groups (*t*_(6)_ = 0.7366, *p *=* *0.4891). Data are mean ± SEM *p <* *0.05 Download Figure 1-1, TIF file.

10.1523/ENEURO.0084-23.2023.tab1-1 Extended Data Table 1-1 Statistical information Download Table 1-1, xlsx file.

EnvA-pseudotyped rabies virus, in which Rgp was replaced with the fluorescent protein mCherry (EnvA-ΔG-MCh), was injected into the dorsal and ventral DG of the same control and runner mice (second injection; Fig. 1*A*) after an interval of six to nine months, resulting in selective infection of OnGC. The rabies virus was then transcomplemented with Rgp provided by RV-SYN-GTRgp, and retrogradely labeled first-order presynaptic neurons (“traced cells”) with mCherry (Fig. 1*A*). The OnGC co-infected by retrovirus and rabies virus, co-expressing GFP and MCh, were considered the origin of the trans-synaptic tracing (“starter cells”), whereas afferent traced cells were distinguished by the expression of MCh only.

The percentage of cells co-expressing GFP and MCh (“starter cells”) per mouse in the control and runner groups was calculated based on the total number of GFP^+^ cells, which increased significantly with running in the dorsal DG (Extended Data Fig. 1-1*B–E*). Only mice with “starter cells” throughout the dorsal-ventral extent of the dentate gyrus with >40% of the total GFP^+^ cells expressing MCh were included in the analysis (four of nine mice in each group). There was no significant difference in the proportion of “starter cells” labeled with respect to the total GFP^+^ cell number between control and runner mice (Extended Data Fig. 1-1*F*), validating that the dual-virus infection (retrovirus-rabies virus) was similar between groups.

Long-term running significantly increased (3.54-fold) the survival of starter cells (Fig. 1*B–D*). This increment was observed in the dorsal (4.5-fold) but not in the intermediate or ventral DG (Fig. 1*E*), consistent with previous studies (Bolz et al., 2015; Vivar et al., 2016). Running also increased the number of presynaptic traced cells (2.04-fold), compared with the control group (Fig. 1*F*). We calculated the ratio of total traced cells to starter cells, the convergence index. Long-term running significantly reduced the ratio of total traced cells to starter cells (Fig. 1*G*). This shows that the running-induced increment in neurogenesis was greater than that of the traced cells (3.5-fold starter cells >2.04-fold traced cells), which may facilitate sparse, nonoverlapping connectivity, supporting the orthogonal coding of neuronal information in the dentate gyrus (Lodge and Bischofberger, 2019). Thus, long-term running may enhance pattern separation ability, a behavior closely linked to adult neurogenesis (Creer et al., 2010), which is among the first deficits indicative of age-related memory decline (Yassa et al., 2010). Furthermore, we identified and quantified traced MCh^+^ cells in each brain area to evaluate adult-born neuron network distribution, as described previously (Vivar et al., 2012, 2016).

### Intrahippocampal network of old adult-born neurons

**Figure 2.**
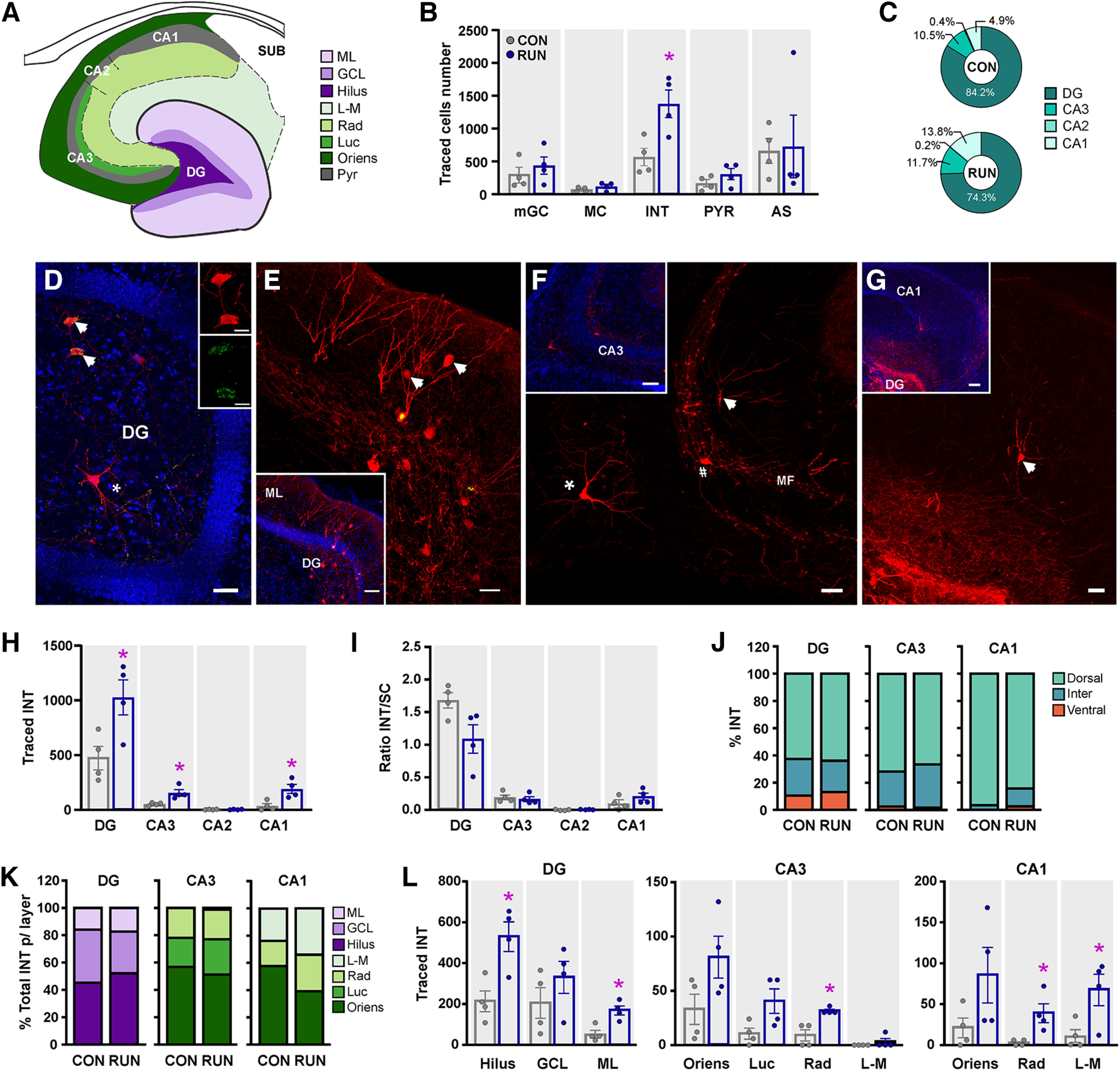
Running increases inhibitory input onto old adult-born neurons. ***A***, Schematic representation of the hippocampal areas and layers. ML, molecular layer; GCL, granule cell layer; L-M, stratum lacunosum moleculare; Rad, stratum radiatum; Luc, stratum lucidum; Oriens, stratum oriens. Pyr, stratum pyramidale. ***B***, Long-term running increases the total number of traced interneurons (INT; *t*_(6)_ = 3.258, *p *=* *0.0173), but not the number of mature granule cells (mGC; *t*_(6)_ = 0.8441, *p *=* *0.4310), mossy cells (MC; *t*_(6)_ = 1.476, *p *=* *0.1904), pyramidal cells (PYR; *t*_(6)_ = 1.348, *p *=* *0.2262), or astrocytes (AS; *t*_(6)_ = 0.1345, *p *=* *0.8974). See Extended Data Figure 2-1 for the innervation ratio and subregional distribution of mGC, MC, and AS. ***C***, Percentage of traced INT per hippocampal area. ***D***, Photomicrograph of the dentate gyrus (DG) showing traced INT and MC. INT (⬆) colabeling MCh^+^ + GABA (red + green; insets). Traced mossy cell expressing MCh only (*). ***E***, Traced INT (⬆) located in the molecular layer (ML) of the dentate gyrus. Inset, Overview of the dentate gyrus. ***F***, Photomicrographs of CA3 area showing mossy fibers (MF) and traced INT located in the stratum oriens (*), lucidum (#), and radiatum (⬆) expressing MCh. Inset, Overview of CA3 area. ***G***, Photomicrograph of area CA1 showing traced INT located in the stratum lacunosum-moleculare (⬆). Inset, Overview of CA1 area. ***H***, Subfield analysis of traced INT number revealed an increase in RUN versus CON in the DG, areas CA3 and CA1, but not in CA2 (*F*_(3,18)_ = 7.005, *p *<* *0.0026). ***I***, The ratio of connectivity between INT and starter cells (INT/SC) was not modified by running (*F*_(1,6)_ = 2.74, *p *>* *0.14). DG INT/SC ratio was higher than that of the other subfields (*F*_(3,18)_ = 6.5, *p *<* *0.036). ***J***, The majority of traced INT are located in the dorsal hippocampus in both CON and RUN mice. ***K***, Percentage of traced INT in the DG, area CA3 and CA1 per layer. ***L***, Analysis within each subfield shows that running increased traced INT number in the DG (*F*_(1,6)_ = 8.13, *p *<* *0.029), area CA3 (*F*_(1,6)_ = 11.79, *p *<* *0.013), and area CA1 (*F*_(1,6)_ = 11.33, *p *<* *0.015). ***D***, Scale bar: 20 μm; inset, 10 μm. Photomicrographs (***E–G***): Scale bar: 50 μm; inset, 100 μm. Nuclei were stained with DAPI (blue). Data are means ± SEM *p <* *0.05.

The intrahippocampal inputs to OnGC derive mainly from the DG, including mature granule cells (mGC), mossy cells (MC), interneurons (INT), and astrocytes (AS), as well as distal intrahippocampal inputs from pyramidal cells (PYR) in middle-aged control and runner mice (Fig. 2*A*,*B*). The total number of traced INT was increased by running (Fig. 2*B*), whereas the quantity and ratio of connectivity of mGC, MC, PYR, and AS was unchanged (Fig. 2*B*; Extended Data Fig. 2-1). Subsequently, we analyzed the regional and subfield distribution of the traced INTs and PYRs in both groups, as there is a differential distribution depending on the subfield (Vivar et al., 2016).

10.1523/ENEURO.0084-23.2023.f2-1 Extended Data Figure 2-1 Old adult-born neurons receive intrahippocampal inputs. ***A***, Photomicrograph of starter old adult-born neurons (SC; red + nuclear yellow) and traced mature GCs (mGCs; red only; arrowhead). ***B***, The ratio of mGC/SC is similar in middle-aged long-term running (RUN) and control (CON) mice (*t*_(6)_ = 1.984, *p *=* *0.0945). ***C***, ***D***, Traced mGCs were located mainly in the dorsal dentate gyrus (dDG) rather than the intermediate (iDG) or ventral (vDG) dentate gyrus and this distribution did not differ between the groups (*F*_(2,12)_ = 0.272, *p *>* *0.77). ***E***, Photomicrograph of traced mossy cells (MC) expressing MCh (red; arrowheads). ***F***, The ratio of MC/SC is not modified by long-term running in middle-aged mice (*t*_(6)_ = 1.408, *p *=* *0.2089). ***G***, ***H***, Traced MC are homogeneously distributed through the dorso-ventral dentate gyrus in middle-aged CON and RUN mice (*F*_(2,12)_ = 0.062, *p *>* *0.94). ***I***, Photomicrograph showing traced astrocytes (red, arrowheads) surrounding the dendritic tree of an old adult-born SC neuron (red + yellow nuclei). ***J***, The ratio of AS/SC was not modified by long-term running (*t*_(6)_ = 1.872, *p *=* *0.1104). ***K***, ***L***, Traced AS are homogeneously distributed through the dorso-ventral dentate gyrus in both groups (*F*_(2,12)_ = 0.744, *p *>* *0.49). Data are mean ± SEM. Scale bar: 10 μm. Nuclei were stained with DAPI (blue). Download Figure 2-1, TIF file.

### Running recruits hippocampal interneurons

As long-term running increases the total number of traced INTs (2.4-fold; Fig. 2*B*), we analyzed their regional distribution. We quantified the traced INT in the DG and areas CA3–CA1 (Fig. 2*A*). The highest percentage of traced INT is located in the DG (CON, 84.2 ± 2.5%; RUN, 74.3 ± 3.2%), followed by long-range INT located in CA3 > CA1 > CA2 (CON, 15.8 ± 2.5%; RUN, 25.6 ± 3.2%) in both middle-aged control and long-term running mice (Fig. 2*C–G*). Running increases the number of traced INT in DG (2.16-fold), CA3 (2.97-fold), and CA1 area (5.33-fold; Fig. 2*H*). These increments are proportional to the increase in starter cells, as the ratio of INT/SC per area is not modified by long-term exercise. DG INT/SC ratio was higher than that of the other subfields (Fig. 2*I*). These INT are mostly located in the dorsal hippocampus in all the areas (Fig. 2*J*).

In the DG, the INT are distributed within the hilus, the granule cell layer (GCL), and molecular layer (ML). In areas CA3 and CA1, the interneurons are mainly located within the *strata oriens*, but are also detected in lucidum (only CA3), radiatum, and lacunosum-moleculare, whereas in CA2 all the INT are located in the stratum oriens (Fig. 2*K*). Running significantly increases the inputs from local INT located in the hilus (2.46-fold) and the molecular layer (3.11-fold) of the DG, while long-range INT increases are observed in the stratum radiatum of areas CA3 (3.5-fold) and CA1 (6.5-fold), and the stratum lacunosum-moleculare (7.5-fold) of area CA1 (Fig. 2*L*). The increase in traced INT may play a role in dampening hippocampal hyperexcitability associated with aging (Lester et al., 2017).

### Running integrates dorsal CA3 pyramidal cells

**Figure 3.**
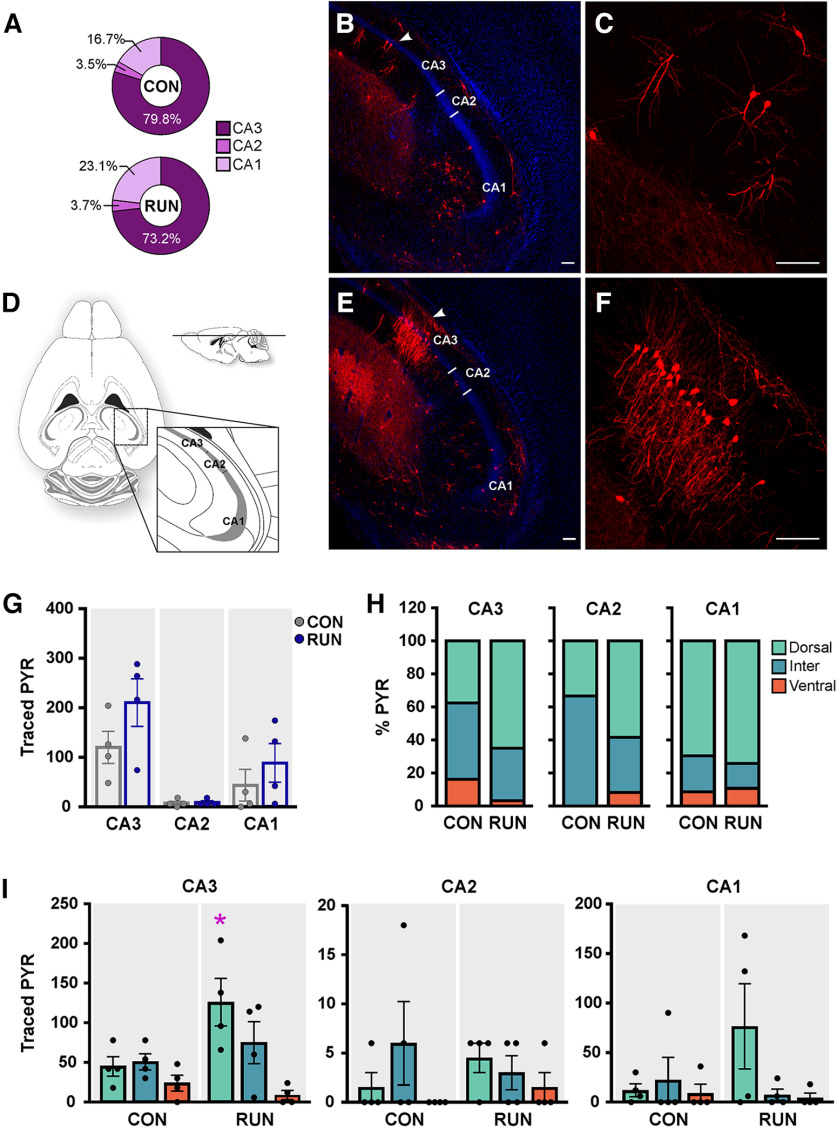
Running increases the connectivity of dorsal CA3 PYR onto old adult-born neurons. ***A***, The percentage of traced pyramidal cells (PYR) was highest in the CA3 area, followed by CA1 and a low percentage in CA2, in both control (CON) and runner mice (RUN). ***B***, ***E***, Photomicrographs of dorsal hippocampal horizontal sections derived from CON (***B***) and RUN (***E***) mice show MCh^+^ PYR (red) located in the CA3 area (arrowhead). ***C***, ***F***, Higher magnification of MCh^+^ PYR in CA3 area from panels (***B***) and (***E***), respectively. ***D***, Schematic representation of a dorsal horizontal brain section (adapted from Paxinos and Franklin, 2007) showing the location of CA3–CA1 areas. ***G***, Long-term running does not modify the number of traced PYR cells in areas CA3–CA1 (*F*_(1,6)_ = 1.84, *p *>* *0.22). ***H***, Percentage of traced PYR in the dorsal, intermediate (inter), and ventral hippocampus in CA3–CA1 areas. ***I***, Dorsal-ventral distribution of analysis of area CA3 revealed that long-term running increases the number of traced PYR in the dorsal area CA3 (*F*_(2,12)_ = 5.37, *p *<* *0.021). There were no changes in the dorso-ventral distribution in area CA2 (*F*_(2,12)_ = 0.97, *p *>* *0.40) or area CA1 (*F*_(2,12)_ = 2.20, *p *>* *0.15). Data are means ± SEM *p <* *0.05. Scale bar: 100 μm. Nuclei were stained with DAPI (blue).

The total number of traced PYR cells was not modified by long-term running (Fig. 2*B*). Distribution analysis in areas CA1–CA3 shows that OnGC receive direct excitatory back-projections from PYR cells mainly from the CA3 area, followed by CA1 and sparse inputs from the CA2 area (Fig. 3*A–F*). Long-term running did not modify the number of traced PYR cells in each area (Fig. 3*G*). We also analyzed the distribution of PYR cells in the dorsal, intermediate, and ventral hippocampus. Traced CA3–CA1 PYR cells are located mainly in the dorsal and intermediate hippocampus under control and long-term running conditions (Fig. 3*H*). Long-term running increased significantly the number of traced PYR cells in the dorsal CA3 area (2.8-fold) only (Fig. 3*B–F*), supporting the idea that CA3 PYR back-projections to OnGC may facilitate the correction of errors produced by serial propagation of cortical information (Lisman et al., 2005).

### Running recruits noncanonical subicular inputs

**Figure 4.**
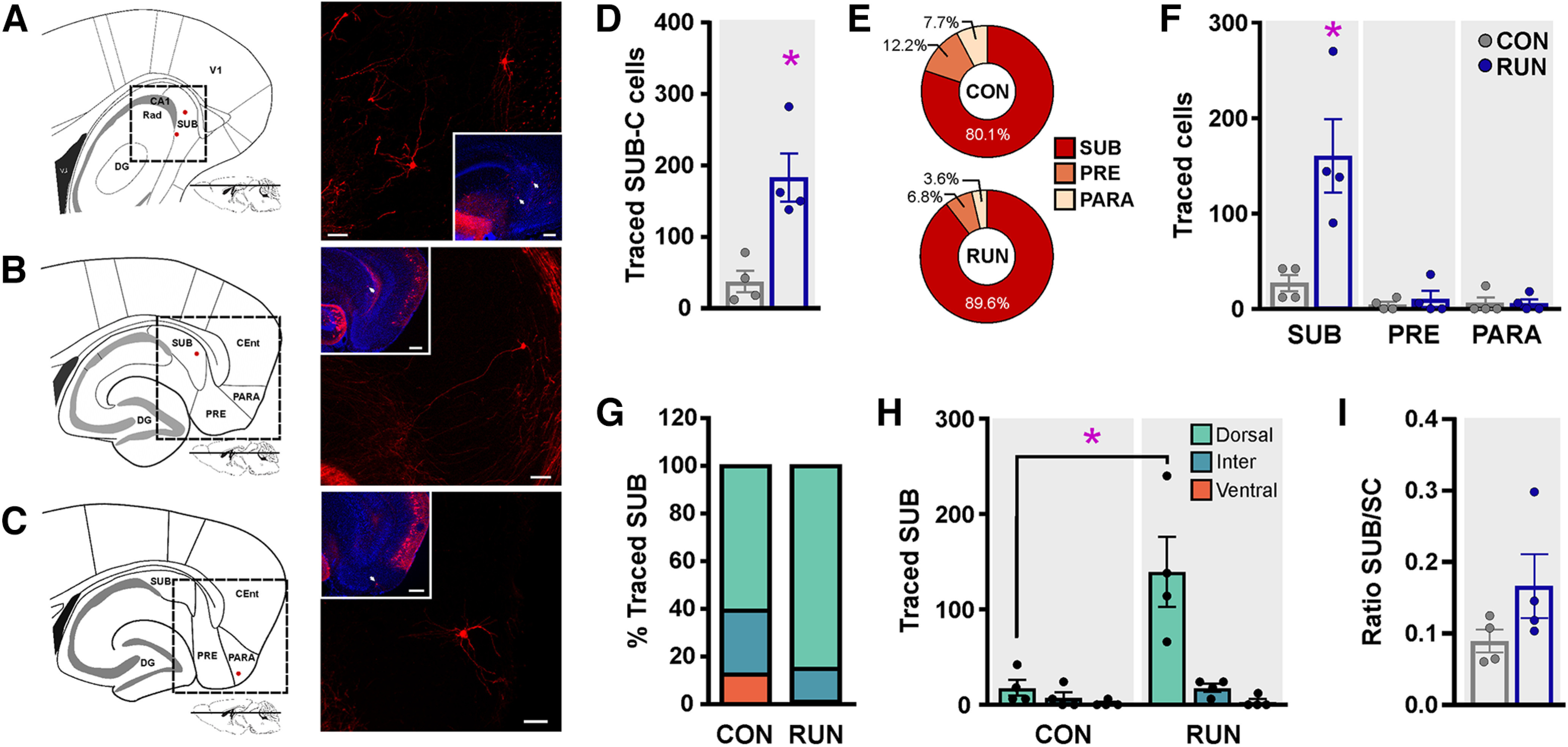
Running recruits noncanonical subicular inputs to old adult-born neuron network. ***A*–*C***, Schematic representations of horizontal slices of the hippocampal formation showing the location of traced cells in the subiculum (SUB; ***A***, ***B***; left) and parasubiculum (PARA, ***C***; left; red dots). Photomicrographs of the SUB (***A***, ***B***, right) and PARA (***C***, right) areas show traced cells expressing MCh. Insets: An overview of the hippocampal formation showing the location of the traced cells of the subicular complex (⬆). Scale bar: 50 μm; inset, 200 μm (***A***, ***B***) and 100 μm (***C***). Nuclei were stained with DAPI (blue). ***D***, Long-term running increases the total number of traced cells in the SUB-C (*t*_(6)_ = 3.979, *p *=* *0.0073). ***E***, Percentage of traced cells in subicular complex areas [SUB, PARA, and presubiculum (PRE)] in control and long-term running mice. ***F***, Long-term running increased SUB traced cell number (*F*_(2,12)_ = 9.42, *p *<* *0.0035). ***G***, The highest percentage of traced cells are located in the dorsal SUB. ***H***, Distribution analysis revealed increased traced cell number in the dorsal SUB with running (*F*_(2,12)_ = 9.28, *p *<* *0.0037). ***I***, The ratio of connectivity between SUB and starter cells (SUB/SC) was not modified by running (*t*_(6)_ = 1.388, *p *=* *0.2145). Data are means ± SEM *p <* *0.05.

The subiculum complex (SUB-C) plays a key role in the mediation of hippocampal-cortical interaction. It is divided into three subdivisions, namely, the subiculum proper (SUB), presubiculum (PRE), and parasubiculum (PARA), with the SUB being the major output of the hippocampus (Witter et al., 1989). Long-term running significantly increased (4.8-fold) the total number of traced cells in the SUB-C (Fig. 4*A–D*). The majority of traced cells are located in the SUB, whereas a small percentage is in the PRE, and PARA in both groups (Fig. 4*E*). Only traced cells in the dorsal SUB (5.94-fold) are increased by running (Fig. 4*F–H*). However, the ratio of SUB/SC (Fig. 4*I*) was unchanged, indicating that the running-induced increase in SUB connectivity correlates with enhanced OnGC survival.

### Perirhinal cortex inputs to OnGC are absent in control mice

**Figure 5.**
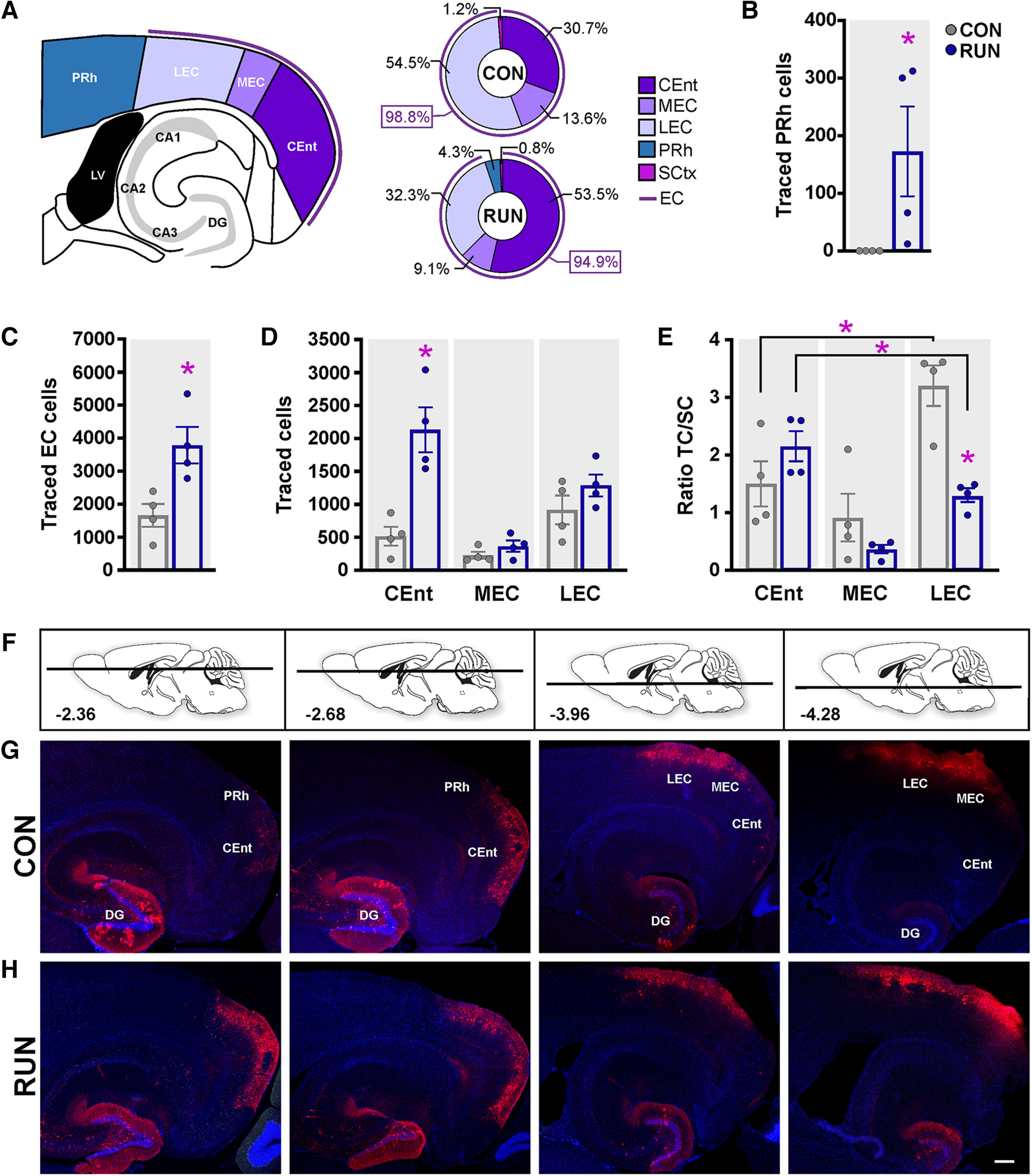
Running preserves PRh input and modifies the distribution of entorhinal cortex afferents onto old adult-born neurons. ***A***, Schematic representation of the cortical input areas in a horizontal slice. Percentages of cortical innervation onto old adult-born neurons in mice housed under control (CON; *n *=* *4) or long-term running (RUN; *n *=* *4) conditions [entorhinal cortex (EC): caudomedial EC (CEnt), lateral EC (LEC) and medial EC (MEC); perirhinal cortex (PRh) and sensory cortices (SCtx)]. ***B***, PRh input to adult-born neurons is only present in RUN mice (Kolmogorov–Smirnov test *p *=* *0.0286). ***C***, Running increases the total number of traced cells in the EC (*t*_(6)_ = 3.234, *p *=* *0.0178). ***D***, Distribution analysis revealed that long-term running increased CEnt traced cell number (*F*_(2,12)_ = 16.031, *p *<* *0.0004). ***E***, Analysis of the TC/SC ratios showed that the CEnt/SC ratio in the CON group is lower than in LEC/SC, whereas in the RUN group the CEnt/SC ratio is higher than the LEC/SC ratio. Running reduced the LEC/SC ratio as compared with the CON group (*F*_(2,12)_ = 19.54, *p *<* *0.002). ***F***, Modified mouse brain atlas images (Paxinos and Franklin, 2007) showing the dorso-ventral horizontal section depth (distance from bregma) corresponding to photomicrographs below. ***G***,***H***, Photomicrographs of horizontal sections showing traced cells (MCh^+^) derived from CON (***G***) and RUN (***H***) mice. Scale bar: 250 μm. Nuclei were stained with DAPI (blue). Extended Data Figure 5-1 depicts the locations and number of sparse inputs from sensory cortices. Data are means ± SEM *p <* *0.05.

The perirhinal cortex (PRh) is important for recognition memory and pattern separation ability (Suzuki and Naya, 2014). In young adult mice, PRh provides input to new neurons (Vivar et al., 2012, 2016). However, PRh connectivity to OnGC is absent in middle-aged control mice. Only entorhinal cortex (EC), and auditory, visual, and sensory cortex (SCtx) input is observed (Fig. 5*A*,*B*; Extended Data Fig. 5-1). Importantly, long-term running preserves the PRh connectivity onto OnGC (Fig. 5*A,B*,*F*,*G*).

10.1523/ENEURO.0084-23.2023.f5-1 Extended Data Figure 5-1 Old adult-born neurons receive sparse inputs from sensory cortices. ***A***, Diagram of a horizontal section (adapted from Paxinos and Franklin, 2007) showing the location of the primary visual cortex (V1), secondary visual cortex (V2), primary auditory cortex (AuD), and primary somatosensory cortex (S1). ***B*** Photomicrographs of a dorsal brain slice showing MCh^+^ cells located in the V1 (⭡), V2 (▲), and AuD (*). ***C***, Long-term running does not modify the inputs from sensory cortices onto the old adult-born neurons (*t*_(6)_ = 0.5774, *p *=* *0.5847). ***D***,***E***, High magnification photomicrographs derived from B showing traced MCh^+^ cells located in (***D***) V1 and V2 and (***E***) AuD. Data are mean ± SEM. Scale bars: 100 μm. Nuclei were stained with DAPI (blue). Download Figure 5-1, TIF file.

### Running modifies CEnt and LEC inputs

From all cortical inputs, the EC is the main cortical input to OnGC in controls (EC 98.8%) and runners (EC 94.9% + PRh 4.3%), and the remainder is a very sparse SCtx (<1.5%) innervation (Fig. 5*A*; Extended Data Fig. 5-1). Running increased the number of traced cells (2.38-fold) in the EC, compared with control conditions (Fig. 5*C*). The EC is considered to be comprised of two major divisions, the lateral entorhinal cortex (LEC) and the medial entorhinal cortex (MEC). The MEC is subdivided into a medial part per se (MEC) and a caudal part (caudomedial entorhinal cortex; CEnt) which are differentially connected to cortical areas and express different neuronal markers and cell types (Insausti et al., 1997; Kobro-Flatmoen and Witter, 2019). Thus, we analyzed the number of traced cells in the LEC, MEC, and CEnt (Fig. 5*A*). In control mice, OnGC received cortical innervation mainly from LEC, which was reflected in a significantly higher ratio of LEC/SC compared with CEnt/SC and MEC/SC (Fig. 5A,*D–F*). Running significantly increased the number of traced cells in the CEnt (4.13-fold), but not in the MEC and LEC (Fig. 5*D*,*G*). CEnt became the main EC input onto OnGC in runners, as the ratio of CEnt/SC is significantly higher than in LEC/SC and MEC/SC (Fig. 5*E*). Thus, long-term running counterbalances the preferential LEC inputs onto OnGC by increasing the CEnt input and thereby complementing the integration of spatial and contextual information.

### Old adult-born neurons retain subcortical connections

**Figure 6.**
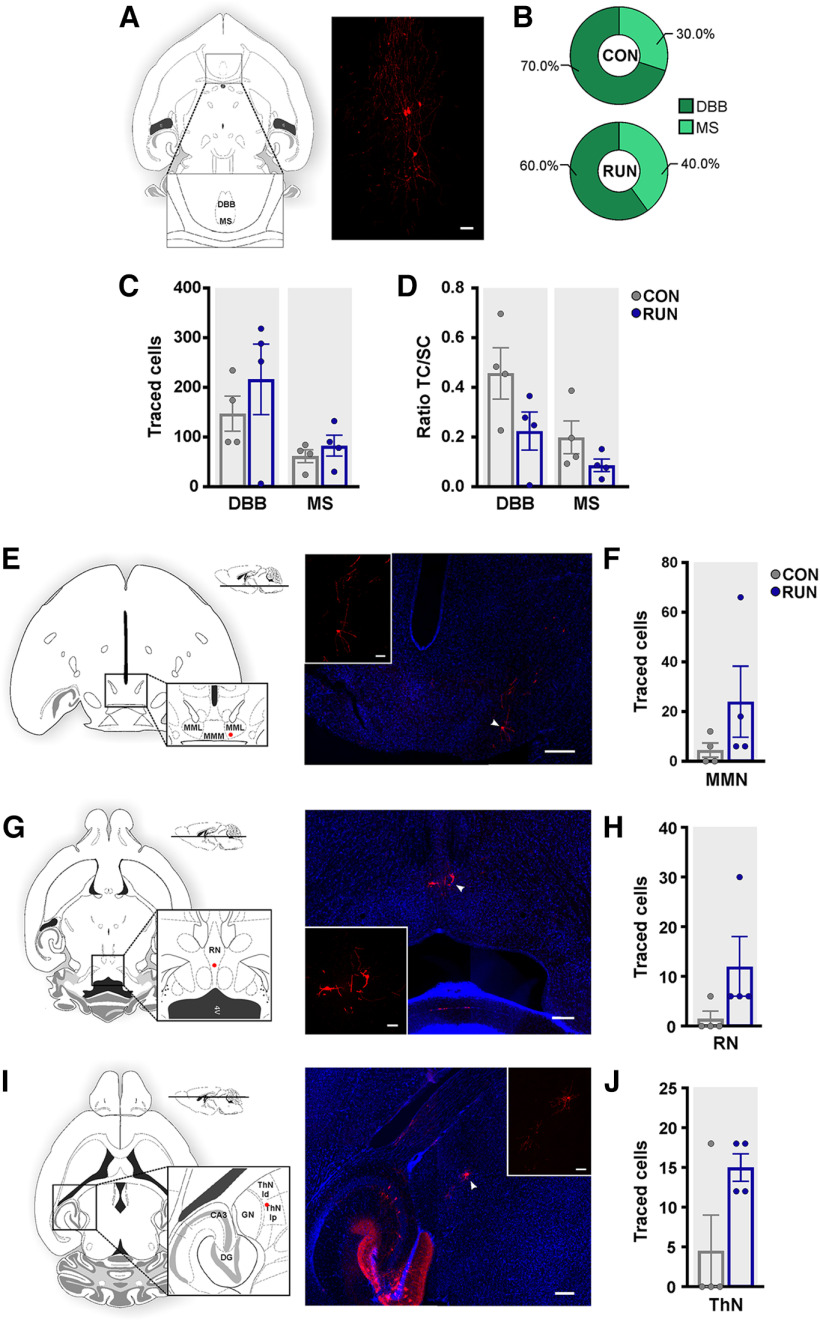
Old adult-born neurons receive inputs from subcortical areas. ***A***, Diagram of a horizontal mouse brain section (adapted from Paxinos and Franklin, 2007) showing the location of the diagonal band of Broca (DBB) and medial septum (MS) of the basal forebrain. Right, Photomicrographs of MCh^+^ cells in the MS. Scale bar: 50 μm. ***B***, The highest percentage of traced cells was located in the DBB. ***C,*** Running did not modify the number of traced cells in either region (*F*_(1,6)_ = 0.61, *p *<* *0.46). ***D***, Analysis of the ratio of TC/SC showed that there was no effect of running (*F*_(1,6)_ = 1.13, *p *>* *0.32). ***E***, ***G***, ***I***, Schematic representation of horizontal mouse brain sections showing the location of MCh^+^ traced cells in the (***E***) medial mammillary nucleus, lateral part (MML), (***G***) Raphe nucleus (RN) and (***I***) thalamic nucleus (ThN; adapted from Paxinos and Franklin, 2007). Right, Photomicrographs of MCh^+^ cells (arrowhead) in the MML (***E***), RN (***G***), and ThN (***I***). The inset shows a higher magnification of MCh^+^ cells in their respective panels. Scale bar: 200 μm; inset 50 μm. Nuclei were stained with DAPI (blue). ***F***, ***H***, ***J***, Running did not modify the number of traced (***F***) medial mammillary nucleus cells (MMN; *t*_(6)_ = 1.309, *p *=* *0.2386), (***H***) RN cells (*t*_(6)_ = 1.698, *p *=* *0.1405), or (***J***) ThN cells (*t*_(6)_ = 2.178, *p *=* *0.0723). Data are means ± SEM *p <* *0.05. MMM, medial mammillary nucleus, medial part; DG, dentate gyrus; GN, dorsal geniculate nucleus; ThN ld, laterodorsal thalamic nucleus; ThN lp, lateral posterior thalamic nucleus.

Basal forebrain inputs to the hippocampus play a relevant role in memory and learning processes (Hasselmo, 1999). Traced cells were observed in the DBB and MS (Fig. 6*A*). Old adult-born neurons receive inputs preferentially from the DBB, under control and running conditions (Fig. 6*B*). However, running did not change the traced cell number nor the ratio of TC/SC of DBB and MS (Fig. 6*C*,*D*). Additionally, sparse traced cells were also observed in the mammillary (MMN; Fig. 6*E*), raphe (RN; Fig. 6*G*), and thalamic (ThN; Fig. 6*I*) nuclei, which were not modified by running (MMN, RN, ThN; Fig. 6*F*,*H*,*J*).

### Long-term running shapes the network of OnGC

**Figure 7.**
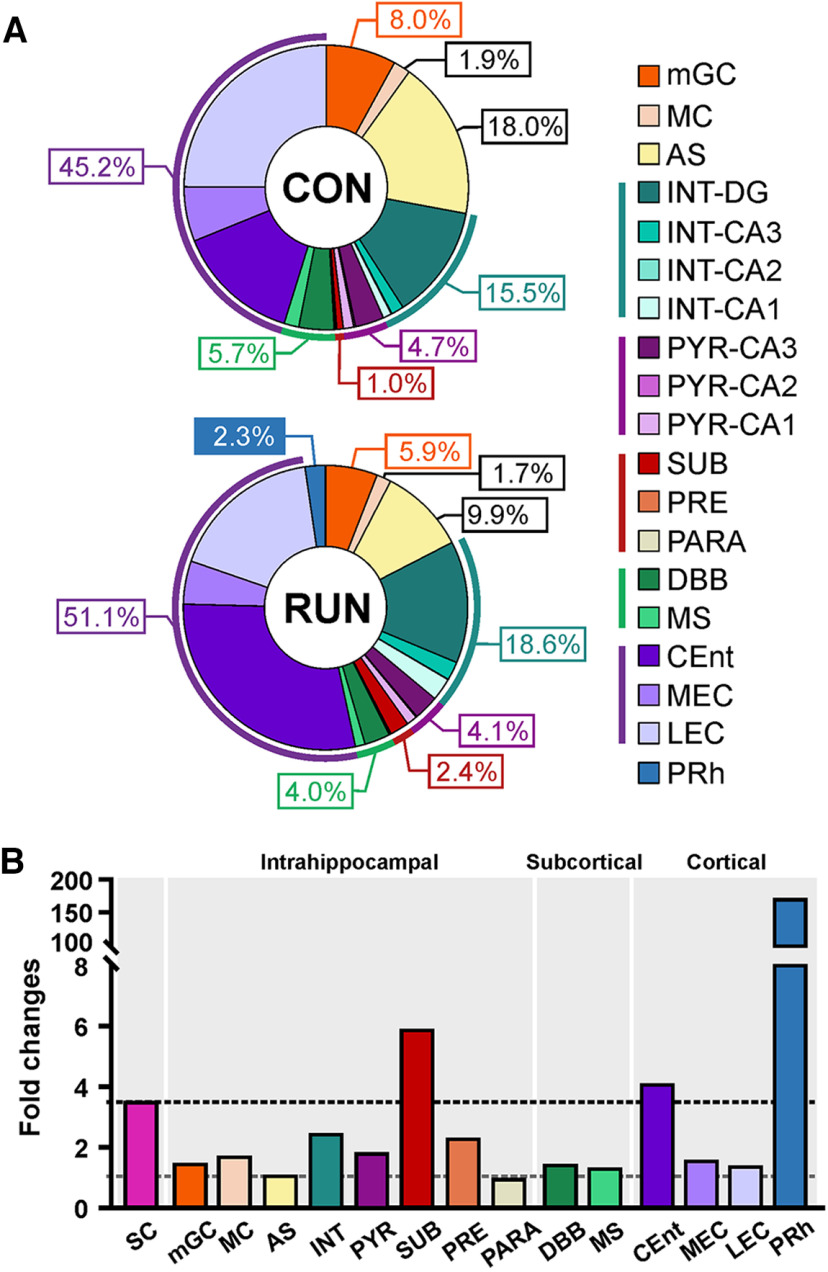
Running throughout middle-age maintains old adult-born neuron wiring. ***A***, Percentages of traced cells in intrahippocampal, cortical, and subcortical areas projecting onto old adult-born neurons in control (CON) and running (RUN) middle-aged mice. ***B***, Long-term running-induced fold changes in old adult-born neurons, starter cells (SC), and in intrahippocampal, cortical and subcortical inputs. Only the subiculum (SUB; ∼6-fold), CEnt (∼4-fold), and PRh exceeded (∼170-fold) the fold change of the starter cells (∼3-fold). mGC, Mature granule cells; MC, Mossy cells; AS, astrocytes; INT, interneurons; PYR, pyramidal cells; PRE, presubiculum; PARA, parasubiculum; DBB, diagonal band of Broca; MS, medial septum; DG, dentate gyrus.

Altogether, our results show that long-term running shaped the connectivity of OnGC by reinforcing inputs from canonical and noncanonical brain regions, such as areas CA1 and CA3, as well as the SUB (Fig. 7*A*,*B*), and increasing INT inputs. At the cortical level, the relative contributions of LEC and CEnt inputs shifted and the PRh input was retained (Fig. 7*A*,*B*). Thus, long-term running throughout middle-age shapes the network of old adult-born neurons in a manner that may benefit contextual and spatial memory.

## Discussion

This study provides evidence that long-term running throughout middle-age increases survival of adult-born granule cells born during early adulthood and reshapes their unique neuronal network. Within the hippocampus, running promotes the recruitment of synaptic inputs to adult-born neurons from interneurons, area CA3 pyramidal cells, and subiculum. Striking changes are observed in cortical inputs. In runners, innervation from the perirhinal cortex, a brain area essential for object recognition, is maintained whereas this input is entirely absent in sedentary control mice. Running also shifts the balance from preferential lateral entorhinal innervation of adult-born neurons to a brain area that is critical for spatial navigation, the caudomedial entorhinal cortex. At the subcortical level, synaptic inputs from the basal forebrain, thalamus, and mammillary nuclei were present but unchanged by running. Altogether, long-term running-induced changes in the network of adult-born neurons may delay or prevent age-related decline in memory function.

Long-term running increases the survival of neurons born during early adulthood in the dorsal hippocampus. Adult-born granule cells are considered to contribute to hippocampus-dependent memory function and display environmentally-induced changes in their neural circuitry (Bergami et al., 2015) during their “critical” time window of high plasticity and excitability, around three to six weeks of cell age (Christian et al., 2020). Thereafter, new neurons reportedly become less excitable and may go for early “retirement” (Alme et al., 2010). In contrast to the retirement hypothesis, other research has shown that new neurons continue to undergo morphological modifications that can be influenced by experience (Lemaire et al., 2012; Cole et al., 2020), and that adult-born neurons older than two months of cell age are recruited during cognitive processes, as they express markers of neuronal activity, such as Arc, cFos, and Zif268 (Erg1) in response to exploration experience or behavioral tasks (Ramirez-Amaya et al., 2006; Kee et al., 2007; Tronel et al., 2015a, b; Ohline et al., 2018). Moreover, nine-month-old adult-born neurons, born during early adulthood (of similar age as the “old” adult-born neurons in the present study), exhibit equally high activity as four-week-old adult-born neurons, measured by Erg1 expression (Ohline et al., 2018). Therefore, long-term running may prevent aging-related memory function decline by increasing the survival and modifying the network of the adult-born neurons born during early adulthood, and by facilitating their participation in cognitive processes. Altogether, long-term running may provide a reserve of plasticity via the old adult-born neurons to the aging brain.

Intrahippocampally, the pattern of connectivity in “old” adult-born neurons in middle-aged mice was retained over time under sedentary control conditions, as traced mature granule cells, interneurons, pyramidal cells, and astrocytes are also observed for young adult-born neurons (Vivar et al., 2012, 2016; Deshpande et al., 2013). This suggests that adult-born neurons have an intrahippocampal network that is basically stable into middle age. This basic microcircuit may display distinctive connectivity depending on the neuron’s birthdate, exhibit specific functions, and be maintained across different stimuli, as has been observed during sequential neurogenesis during embryonic development (Deguchi et al., 2011; Cossart and Khazipov, 2022; Huszár et al., 2022).

Old adult-born neurons mainly receive local DG interneuron input, consistent with observations in young adult mice (Markwardt et al., 2011; Vivar et al., 2012, 2016; Groisman et al., 2020); however, long-range interneurons from areas CA3–CA1 also contribute significantly to their inhibitory network. Indeed, inhibitory long-range back-projections to the DG may play a key role in sharp-wave-associated episodic memory sequencing and replay (Szabo et al., 2017). Unique to the old adult-born neurons is the observed running-induced increased innervation from dorsal area CA3–CA1 and DG interneurons. Indeed, in young mice, six weeks of running did not increase interneuron innervation and resulted in a decrease in the ratio of traced interneuron to starter cells (Vivar et al., 2016). The current finding is particularly important as in aging subjects there is an increase in DG/CA3 hyperexcitability which is associated with age-dependent memory loss (Yassa et al., 2011; Koh et al., 2013; Haberman et al., 2017). Enhanced interneuron innervation of old adult-born neurons may mitigate intrahippocampal hyperexcitability and thereby improve memory function.

Pyramidal cells are among the earliest excitatory glutamatergic intrahippocampal inputs to developing adult-born neurons (Sah et al., 2017). The direct excitatory back-projection from pyramidal cells is initially from area CA1, however, when adult-born neurons are mature input mainly derives from area CA3 pyramidal cells (Vivar et al., 2012, 2016; Sah et al., 2017). While the total number of pyramidal cell inputs was not changed, our distribution analysis showed a substantial running-induced increase in back-projecting neurons from the dorsal CA3 area. Previous studies have shown that pyramidal cells, preferentially located in the ventral hippocampus, innervate DG granule cells; however, those findings were not specific to inputs to adult-born neurons (Li et al., 1994). The pattern of pyramidal neuron innervation to adult-born dentate granule cells in young (Vivar et al., 2016) and middle-aged mice indicates that the dorsal aspect is dominant. This back-projection to the adult-born neurons may be a mechanism for encoding sequential information. Thus, old adult-born neurons may facilitate the correction of errors, via pyramidal cell back-projections, produced by serial propagation of cortical information (Lisman et al., 2005).

The subiculum is considered the major output of the hippocampus (Witter et al., 1989). However, previous studies have shown that the subiculum back-projects to the dentate gyrus as well as area CA1 (Deshpande et al., 2013; Vivar et al., 2016; Xu et al., 2016). Specifically, in the dentate gyrus, young adult-born neurons receive sparse direct back-projection from the subicular complex, which is not modified by running (Deshpande et al., 2013; Vivar et al., 2016). In the present study, we show that running substantially increases the back-projection from the dorsal subiculum onto old adult-born granule cells. This connectivity may provide navigation-associated information (Kitanishi et al., 2021) and mediate the long-term running-induced improvement in spatial memory function (Marlatt et al., 2012; Vivar et al., 2012).

Running also impacts the cortical network of old adult-born neurons. The perirhinal cortex, a brain area essential for object recognition (Bussey et al., 2005), is considered to project to the hippocampus indirectly via the lateral entorhinal cortex and directly to area CA1 (Sethumadhavan et al., 2022). Input to the dentate gyrus has been considered controversial (Liu and Bilkey, 1997; Witter et al., 1999); however, in young adult mice, there are direct projections to adult-born neurons (Vivar et al., 2012, 2016). In addition, a lesion of the perirhinal and lateral entorhinal cortex impairs pattern separation (Vivar et al., 2012; Voss et al., 2019). Here, we show that perirhinal connectivity to old adult-born neurons is absent in middle-aged control mice but is preserved by running. This may help resolve the controversy as to whether there is a perirhinal input to the DG, because when animals are older the maintenance may require exercise. Direct input to adult-born neurons may be a unique source of cortical information that is highly relevant to pattern separation ability (Kent et al., 2016; Stevenson et al., 2020). Indeed, aging-related memory function decline is associated with the degradation of synaptic inputs from the perirhinal and entorhinal cortex onto the hippocampus (Yassa et al., 2010, 2011; Montchal et al., 2019; Stevenson et al., 2020). Our results show that running not only rescued perirhinal connectivity but also increased and altered the relative contribution of the lateral and caudomedial entorhinal cortices to the network of old adult-born neurons. In adult control rodents, the lateral entorhinal cortex is the main input to adult-born neurons (Vivar et al., 2012, 2016; Woods et al., 2018), and we observed a similar pattern in the middle-aged mice. Under running conditions, however, the caudomedial input greatly expands and counterbalances the preferential lateral entorhinal inputs thereby enhancing the integration of spatial and contextual information, which may delay or prevent age-related memory decline.

A limitation of this study is the absence of further electrophysiological and behavioral studies to better understand the precise contribution of traced brain areas and cell types to the function of “old” adult-born granule cells. Indeed, the TVA-EnvA tracing system we used indicates the presence of synaptic contacts between inputs and adult-born neuron starter cells, but future functional studies are needed to complement the present experiments. The system only labels a fraction of inputs to starter cells. In addition, each input cell may make multiple synapses onto a target cell (Feldmeyer et al., 2002, 2006). Furthermore, rabies glycoprotein expression levels in the starter cells, the number and location of synaptic contacts, the subcellular locations of synaptic contacts, or receptors in the axon terminals may differ between input cell types (Callaway and Luo, 2015; Rogers and Beier, 2021).

Overall, long-term exercise profoundly benefits the aging brain. We show that chronic physical activity from young adulthood into middle age maintains and enhances the network that innervates adult-born neurons. While these findings are limited to the circuitry of adult-born neurons we expect that they are representative and indicative of the effects of running on the brain as a whole and provide novel insight as to how exercise helps maintain memory function during aging.

## Synthesis

Reviewing Editor: Karyn Frick, University of Wisconsin-Milwaukee Decisions are customarily a result of the Reviewing Editor and the peer reviewers coming together and discussing their recommendations until a consensus is reached. When revisions are invited, a fact-based synthesis statement explaining their decision and outlining what is needed to prepare a revision will be listed below. The following reviewer(s) agreed to reveal their identity: NONE. Note: If this manuscript was transferred from JNeurosci and a decision was made to accept the manuscript without peer review, a brief statement to this effect will instead be what is listed below.

This revised manuscript was transferred from the Journal of Neuroscience. The two reviewers of the original submission agreed to re-review the manuscript for eNeuro. Reviewer 1 felt that the authors had sufficiently addressed their comments. However, Reviewer 2 felt that their concerns about statistical methods and CA2/3 location were not sufficiently addressed, which we have discussed via the Review Consultation. What follows below are the author‘s response and reviewer’s response, followed by my requests for revision.

STATISTICS:

Author Response: We thank the reviewer for the comment, however, our statistics are appropriate.

Reviewer 2: I realize that there are divergent views on statistical approaches and I don’t think there is a one size fits all approach to this. But correcting for multiple comparisons is far from unusual and is widely accepted as an important step towards reducing false positive error rates, which are common when low sample sizes are used (such as n=4 in this study). Can the authors explain why not correcting is appropriate? Some statistically significant effects would become nonsignificant. This is not necessarily a problem, however the authors would have to change their interpretations. Why is it appropriate to use parametric tests for non-normally distributed data? Wouldn’t is also be appropriate, or even more appropriate, to use non parametric tests? These issues tie in to questions I had below where the authors attempt to make comparisons with previous work that used short term running. If some of the effects presented here are statistically questionable then how can we even attempt to make further comparisons with a previous study?

Editor: I agree with the reviewer’s concern about your exclusive reliance on t-tests (33 tests from what I can tell) without any kind of correction for false positives. I see one of three possible solutions: 1) include at least some ANOVAs on regional or subregional data with posthocs to test between-group differences, 2) employ a correction method for existing t-tests to reduce the likelihood of type 1 error, or 3) keep all t-tests as is but add meaningful discussion to the statistical methods as to why ANOVAs or corrections are not appropriate for these data and how your approach addresses type 1 errors. Regarding ANOVAs, there are data sets where it seems reasonable that you could use ANOVA instead of multiple t-tests (e.g., Fig. 2L, Fig. 3 G,I). ANOVAs followed by posthocs (e.g., Tukey, Neuman-Keuls) would lend more statistical power to your analyses and would permit you to make additional valuable conclusions. For example, in Fig. 2L an ANOVA may support the conclusion there’s a significant main effect of running across subregions of the DG, that there’s a significant main effect of subregion on traced INT number, and that perhaps there’s a significant interaction between treatment and subregion. Although you may not have originally intended to examine subregional differences in this way, such information adds to the paper in my view. Thus, I would suggest conducting ANOVAs on regional or subregional data sets (i.e., Fig. 2H, 2I, 2L, 3G, 3I, 4F, 4H, 5D, 5E, 6C, 6D) that include posthoc tests to assess between group differences instead of dozens of uncorrected t-tests. If such ANOVAs are not appropriate for your hypothesis, then you could select option 2 and use Bonferroni corrections or some other method to reduce the likelihood of false positive errors in your t-tests. If you do not select options 1 or 2, then you must provide justification in the manuscript for why the statistical approach you used is appropriate for both testing your hypotheses and ensuring low type 1 error.

CA2/CA3 IN FIG. 3:

Author Response: We appreciate the suggestion of the reviewer to utilize a CA2-specific marker (e.g. RGS14, Evans et al., 2014, doi: 10.1002/cne.23395). We will certainly include this in future studies. The dense cells depicted in Figure 3 are area CA3 cells based on the adjacent brain atlas (Paxinos and Franklin, 2007) map.

Reviewer 2: When I look at the atlas image in Fig 2D and compare it to the histological image in Fig 2E it looks to me like these red cells are precisely in the area that the atlas identifies as CA2. With a complete absence of cells on either side (ie in CA1 and CA3). I don’t see how this can be interpreted as anything else. I am open to the possibility that I may be wrong about this, but I think this is a legitimate concern that should be seriously considered and not be relegated to future studies. From my perspective, proceeding “as is” would result in a paper with a clear and obvious misinterpretation. If the authors do not have tissue to do this staining, then I still think they could/should interpret these cells as CA2 cells simply based on anatomical position.

Editor: I appreciate that it can difficult to extrapolate locations from a schematic diagram to a representative fluorescent image. To help the reviewer (and other readers) better reconcile the two, I suggest adding dashed lines to the fluorescent images in Fig 2B and 2E to indicate what you consider to be the divisions between CA1, 2, and 3 in these particular images.

## Author Response

Response to the Editor and Reviewer 2

STATISTICS:

Reviewer 2: I realize that there are divergent views on statistical approaches and I don’t think there is a one size fits all approach to this. But correcting for multiple comparisons is far from unusual and is widely accepted as an important step towards reducing false positive error rates, which are common when low sample sizes are used (such as n=4 in this study). Can the authors explain why not correcting is appropriate? Some statistically significant effects would become nonsignificant. This is not necessarily a problem, however the authors would have to change their interpretations. Why is it appropriate to use parametric tests for non-normally distributed data? Wouldn’t is also be appropriate, or even more appropriate, to use non parametric tests? These issues tie in to questions I had below where the authors attempt to make comparisons with previous work that used short term running. If some of the effects presented here are statistically questionable then how can we even attempt to make further comparisons with a previous study?

Editor: I agree with the reviewer’s concern about your exclusive reliance on t-tests (33 tests from what I can tell) without any kind of correction for false positives. I see one of three possible solutions: 1) include at least some ANOVAs on regional or subregional data with posthocs to test between-group differences, 2) employ a correction method for existing t-tests to reduce the likelihood of type 1 error, or 3) keep all t-tests as is but add meaningful discussion to the statistical methods as to why ANOVAs or corrections are not appropriate for these data and how your approach addresses type 1 errors. Regarding ANOVAs, there are data sets where it seems reasonable that you could use ANOVA instead of multiple t-tests (e.g., Fig. 2L, Fig. 3 G,I). ANOVAs followed by posthocs (e.g., Tukey, Neuman-Keuls) would lend more statistical power to your analyses and would permit you to make additional valuable conclusions. For example, in Fig. 2L an ANOVA may support the conclusion there’s a significant main effect of running across subregions of the DG, that there’s a significant main effect of subregion on traced INT number, and that perhaps there’s a significant interaction between treatment and subregion. Although you may not have originally intended to examine subregional differences in this way, such information adds to the paper in my view. Thus, I would suggest conducting ANOVAs on regional or subregional data sets (i.e., Fig. 2H, 2I, 2L, 3G, 3I, 4F, 4H, 5D, 5E, 6C, 6D) that include posthoc tests to assess between group differences instead of dozens of uncorrected t-tests. If such ANOVAs are not appropriate for your hypothesis, then you could select option 2 and use Bonferroni corrections or some other method to reduce the likelihood of false positive errors in your t-tests. If you do not select options 1 or 2, then you must provide justification in the manuscript for why the statistical approach you used is appropriate for both testing your hypotheses and ensuring low type 1 error.

Response: Thank you for the comments on our statistical analysis. We understand the concerns about a possible Type 1 error. However, in this study 2 groups were examined, control (CON) and runner (RUN). Unpaired t-tests are appropriate to compare between two independent groups. That said, we highlight that we analyzed multiple variables (not multiple groups) using an experimental design with One Fixed Factor (Treatment) containing only two levels (CON and RUN). If there were multiple groups (three or more) and repeated t-tests had been applied without correction, then there would be a concern of Type 1 Error. 2

We do appreciate the recommendation to use ANOVAs for the subregional analysis as this increases the power of these analyses. We have modified the text accordingly to indicate we used two-way ANOVA (Treatment x Region) with repeated measures, followed by posthoc Tukey test. CON vs RUN pairwise comparison for each region was performed when an interaction between factors was identified.

The reviewer raised concerns about the normal distribution of the data. Indeed, with N= 4/group we cannot accurately determine this, however, parametric statistics such as t-test and ANOVA provide more statistical power, as well as an estimation of the magnitude of the effect. In fact, when the sample sizes are N=4/group, the Mann-Whitney test (a nonparametric alternative to Unpaired t-test) does not have power to identify any differences in cases in which there is at least one overlap between group data points. Therefore, this alternative would increase the chance of Type 2 Error. We did use the two sample

Kolmogorov-Smirnov nonparametric test in one instance for CON vs RUN comparison when one of the groups presented only ZERO as values (Figure 5B). The full statistical scores are listed in Table 1. CA2/CA3 IN FIG. 3:

Reviewer 2: When I look at the atlas image in Fig 2D and compare it to the histological image in Fig 2E it looks to me like these red cells are precisely in the area that the atlas identifies as CA2. With a complete absence of cells on either side (ie in CA1 and CA3). I don’t see how this can be interpreted as anything else. I am open to the possibility that I may be wrong about this, but I think this is a legitimate concern that should be seriously considered and not be relegated to future studies. From my perspective, proceeding “as is” would result in a paper with a clear and obvious misinterpretation. If the authors do not have tissue to do this staining, then I still think they could/should interpret these cells as CA2 cells simply based on anatomical position.

Editor: I appreciate that it can be difficult to extrapolate locations from a schematic diagram to a representative fluorescent image. To help the reviewer (and other readers) better reconcile the two, I suggest adding dashed lines to the fluorescent images in Fig 2B and 2E to indicate what you consider to be the divisions between CA1, 2, and 3 in these particular images.

Response: Thank you very much for this helpful suggestion. We have now modified Figure 3B and Figure 3E to clearly delineate the borders between the CA areas.
