# Supplementary figures and images for: Running throughout Middle-Age Keeps Old Adult-Born Neurons Wired
Source: eNeuro. 2023 May 17;10(5):ENEURO.0084-23.2023. doi: 10.1523/ENEURO.0084-23.2023 (PMC10217125; doi:10.1523/ENEURO.0084-23.2023)

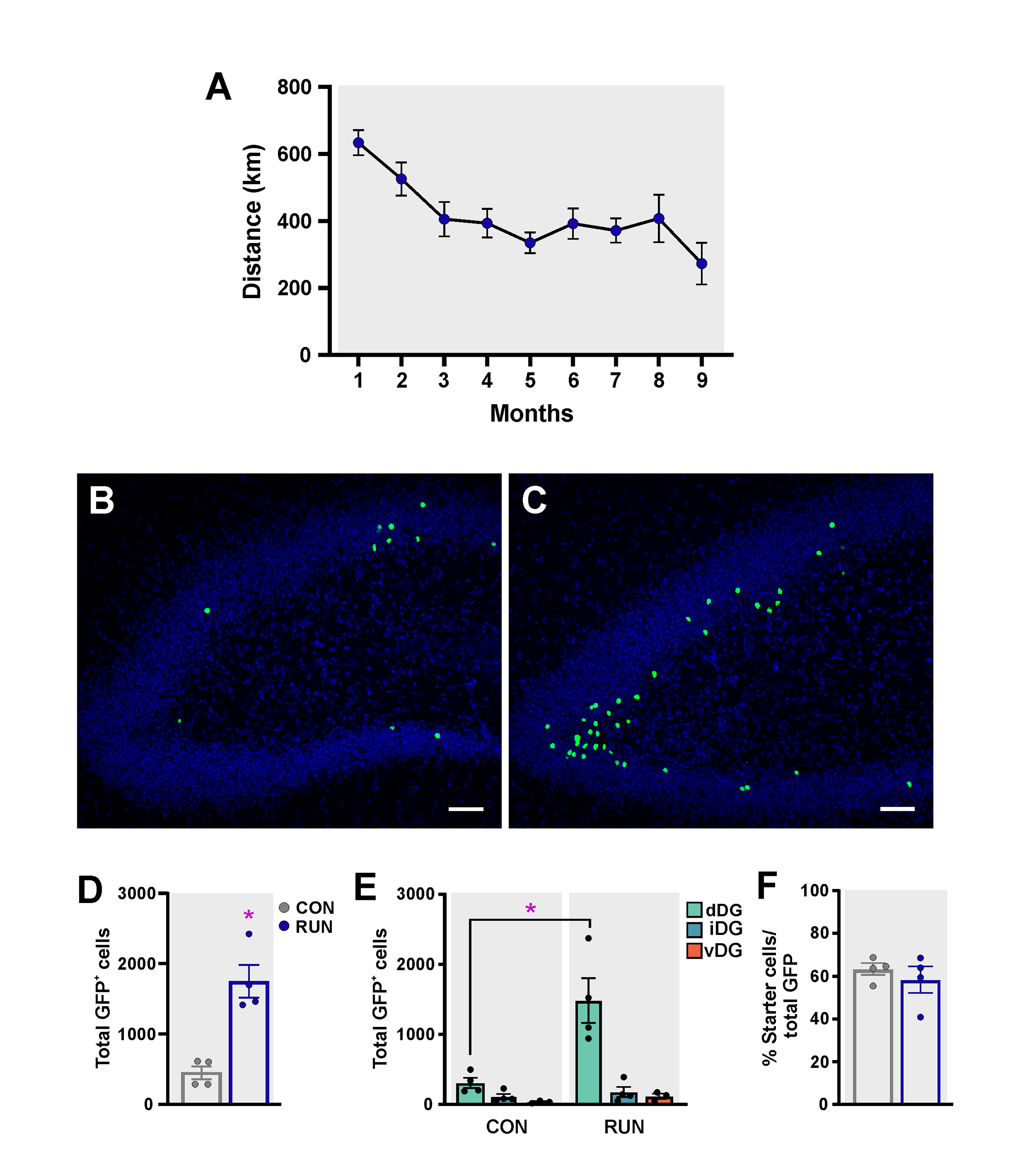

Supplement: Extended Data Figure 1-1 — Running increases the survival of old adult-born neurons born during early adulthood. A, Long-term running distance, there is a significant reduction in monthly distance run over time in male C57Bl/6 mice (n = 9; F(1,7) = 12.86, p < 0.0089). B, C, Photomicrographs of the dorsal dentate gyrus showing GFP+ cells from control (CON; B) and long-term running mice (RUN; C). Scale bar: 50 μm. Nuclei were stained with DAPI (blue). D, Long-term running increases the number of total GFP+ cells in the dentate gyrus compared to control middle-aged mice (t(6) = 5.196, p = 0.002; CON, n = 4; RUN, n = 4). E, Dorsal to ventral distribution analysis shows that running increases the number of GFP+ cells in the dorsal (dDG) but not intermediate (iDG) or ventral (vDG) dentate gyrus (F(2,12) = 8.64, p < 0.0047). F, The proportion of double-labeled cells (starter cells) with respect to the total number of GFP+ cells is similar between groups (t(6) = 0.7366, p = 0.4891). Data are mean ± SEM *p < 0.05 Download Figure 1-1, TIF file. [file enu-eN-NWR-0084-23-s01.tif]

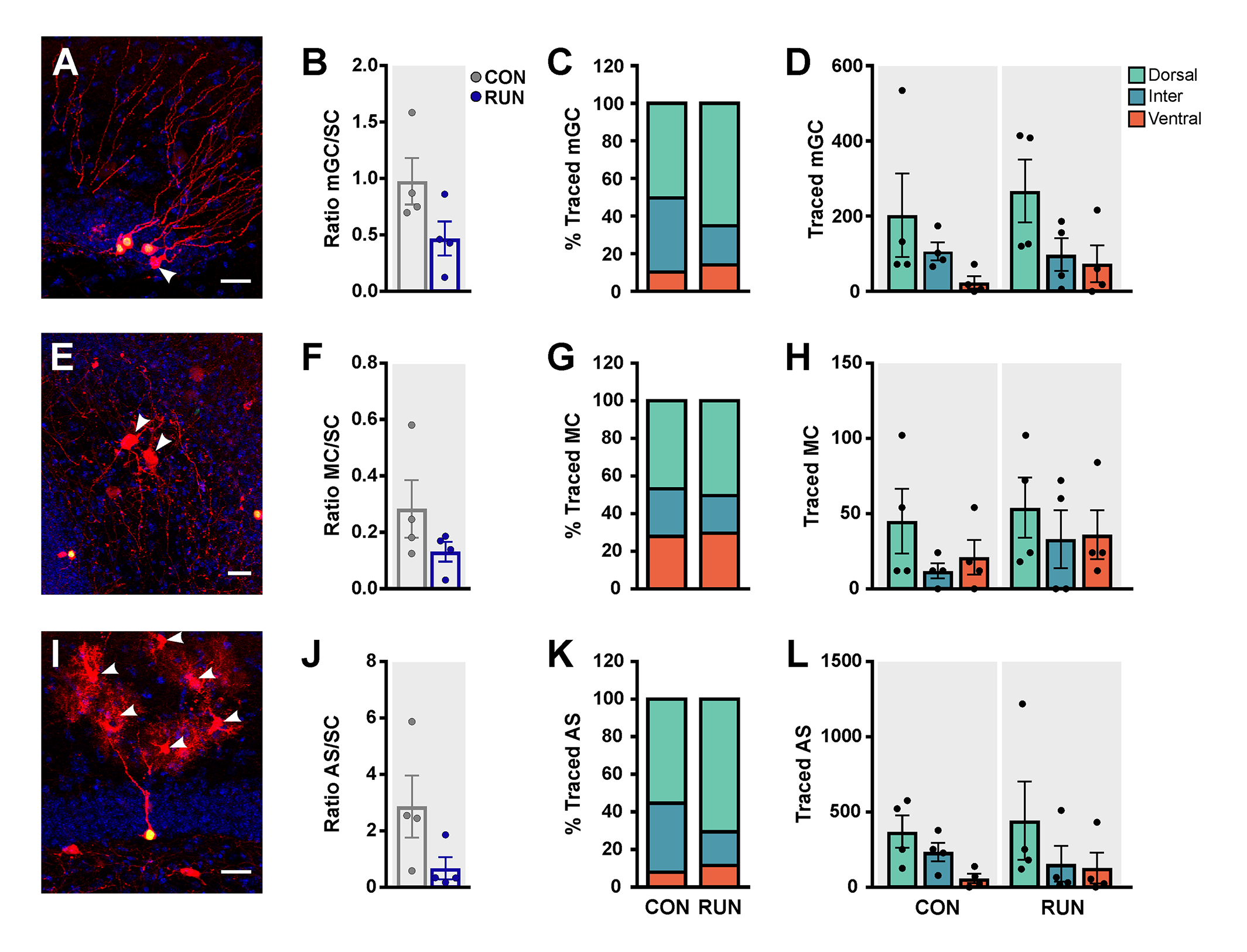

Supplement: Extended Data Figure 2-1 — Old adult-born neurons receive intrahippocampal inputs. A, Photomicrograph of starter old adult-born neurons (SC; red + nuclear yellow) and traced mature GCs (mGCs; red only; arrowhead). B, The ratio of mGC/SC is similar in middle-aged long-term running (RUN) and control (CON) mice (t(6) = 1.984, p = 0.0945). C, D, Traced mGCs were located mainly in the dorsal dentate gyrus (dDG) rather than the intermediate (iDG) or ventral (vDG) dentate gyrus and this distribution did not differ between the groups (F(2,12) = 0.272, p > 0.77). E, Photomicrograph of traced mossy cells (MC) expressing MCh (red; arrowheads). F, The ratio of MC/SC is not modified by long-term running in middle-aged mice (t(6) = 1.408, p = 0.2089). G, H, Traced MC are homogeneously distributed through the dorso-ventral dentate gyrus in middle-aged CON and RUN mice (F(2,12) = 0.062, p > 0.94). I, Photomicrograph showing traced astrocytes (red, arrowheads) surrounding the dendritic tree of an old adult-born SC neuron (red + yellow nuclei). J, The ratio of AS/SC was not modified by long-term running (t(6) = 1.872, p = 0.1104). K, L, Traced AS are homogeneously distributed through the dorso-ventral dentate gyrus in both groups (F(2,12) = 0.744, p > 0.49). Data are mean ± SEM. Scale bar: 10 μm. Nuclei were stained with DAPI (blue). Download Figure 2-1, TIF file. [file enu-eN-NWR-0084-23-s02.tif]

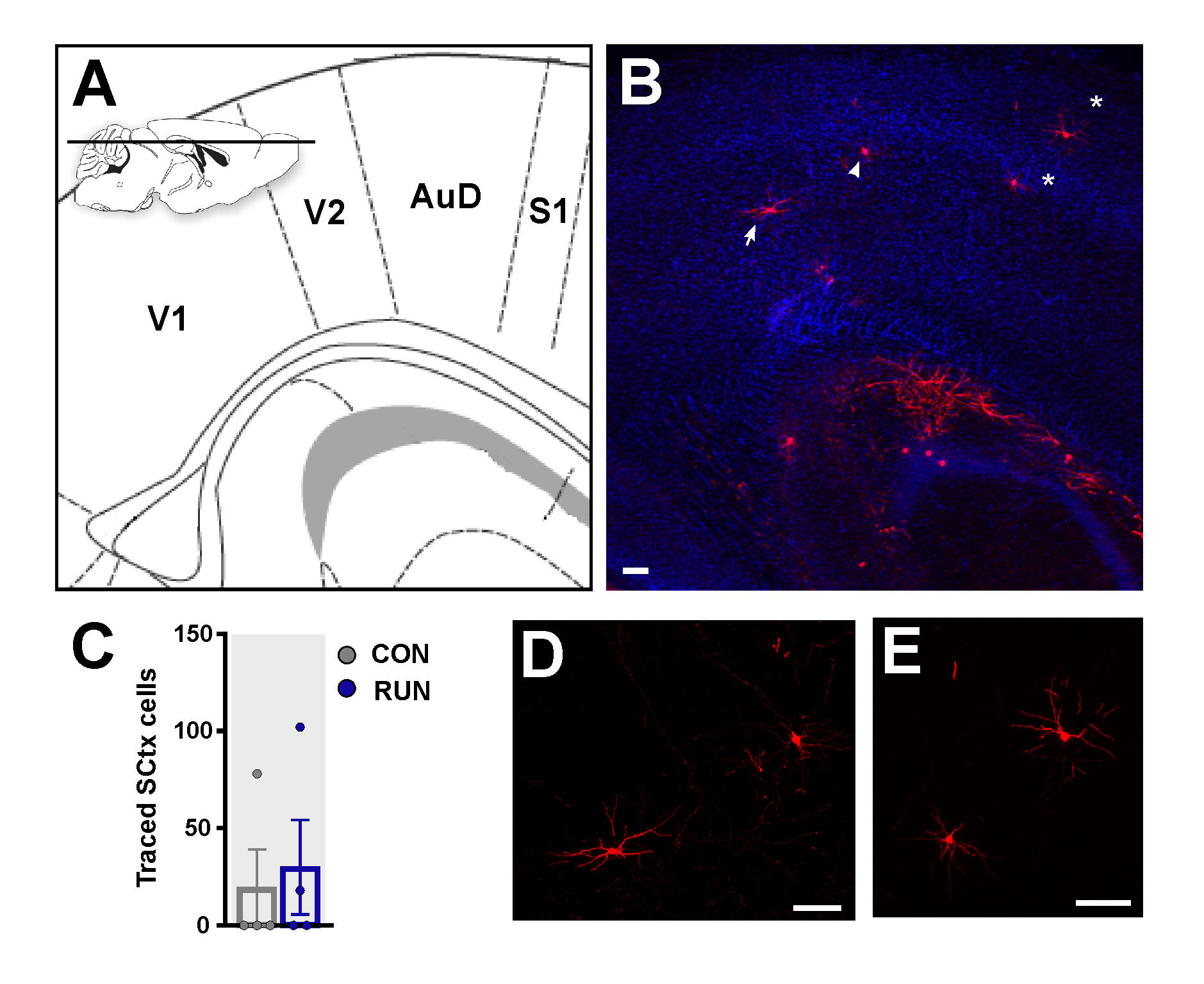

Supplement: Extended Data Figure 5-1 — Old adult-born neurons receive sparse inputs from sensory cortices. A, Diagram of a horizontal section (adapted from Paxinos and Franklin, 2007) showing the location of the primary visual cortex (V1), secondary visual cortex (V2), primary auditory cortex (AuD), and primary somatosensory cortex (S1). B Photomicrographs of a dorsal brain slice showing MCh+ cells located in the V1 (⭡), V2 (▲), and AuD (*). C, Long-term running does not modify the inputs from sensory cortices onto the old adult-born neurons (t(6) = 0.5774, p = 0.5847). D,E, High magnification photomicrographs derived from B showing traced MCh+ cells located in (D) V1 and V2 and (E) AuD. Data are mean ± SEM. Scale bars: 100 μm. Nuclei were stained with DAPI (blue). Download Figure 5-1, TIF file. [file enu-eN-NWR-0084-23-s03.tif]
